# Sulfadiazine-Induced Obstructive Nephropathy Presenting with Upper Urinary Tract Extravasation

**DOI:** 10.1089/cren.2016.0093

**Published:** 2016-09-01

**Authors:** Maharan Kabha, Snir Dekalo, Sophie Barnes, Ishay Mintz, Haim Matzkin, Mario Sofer

**Affiliations:** ^1^ Department of Urology, Tel-Aviv Sourasky Medical Center, Affiliated to the Sackler Faculty of Medicine, Tel-Aviv University, Tel-Aviv, Israel.; ^2^ Department of Radiology, Tel-Aviv Sourasky Medical Center, Affiliated to the Sackler Faculty of Medicine, Tel-Aviv University, Tel-Aviv, Israel.

**Keywords:** sulfadiazine, obstructive uropathy, extravasation, hydronephrosis

## Abstract

***Background:*** Obstructive nephropathy is an uncommon side effect of sulfadiazine, which is used for the treatment of toxoplasmosis. We present a case of acute renal colic and urine extravasation of a patient shortly after she was started on this medication.

***Case Presentation:*** A 31-year-old female presented with acute renal colic 2 weeks after starting treatment with sulfadiazine and pyrimethamine for ocular toxoplasmosis.

***Results:*** A noncontrast computed tomography revealed left hydronephrosis and fluid located around the kidney and in the left gutter. There were no urinary stones. Administration of intravenous contrast revealed significant urine extravasation at the level of the ureteropelvic junction. Intravenous contrast injection confirmed that the extravasation consisted of urine leakage at the ureteropelvic junction. Her clinical condition improved with the insertion of an internal stent, which was left in place for 4 weeks. A retrograde pyelography performed at the time of the internal stent removal ruled out persistent extravasation and filling defects in the left upper urinary tract. Considering the clinical circumstances and the imaging results, it appears that this is a first reported case of sulfadiazine-induced obstructive uropathy associated with urine extravasation.

***Conclusion:*** Although rare, obstructive uropathy related to sulfadiazine medication should be promptly suspected, diagnosed, and treated. Patients should be instructed to substantially increase their liquid intake while on that medication.

## Introduction and Background

Sulfadiazine combined with pyrimethamine and steroids is considered the “classic” treatment for ocular toxoplasmosis.^1^ Obstructive uropathy induced by sulfadiazine medication is rare and has been occasionally described.^2,3^ We now report a case of sulfadiazine-associated urinary extravasation, a finding that, to the best of our knowledge, has not been previously reported.

## Presentation of Case

**FIG. 1.**
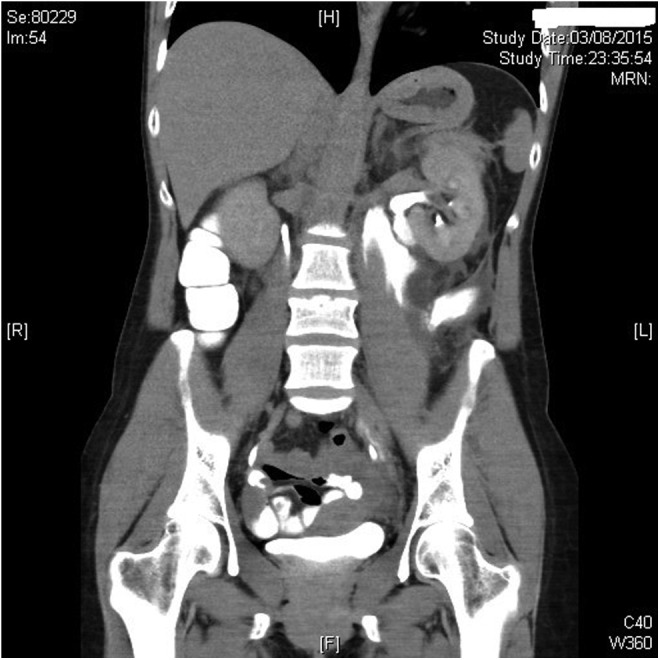
Coronal reconstruction of a computerized tomographic urography showing bilateral renal excretion with left hydronephrosis and urine extravasation from the ureteropelvic junction site.

A 31-year-old female patient presented to the emergency room with left renal colic. She appeared to be in considerable distress associated with bouts of excruciating left flank pain, nausea, and vomiting. The patient was afebrile, her blood pressure within normal limits, and her heart rate was 80 beats per minute. Physical examination revealed severe left flank and abdominal tenderness without signs of peritonitis. Her laboratory test results were hemoglobin 13.4 g/dL, leukocytosis 16.100/mm^3^, mild renal failure with a creatinine level of 1.2 mg/dL, and microhematuria without signs of infection in the urinalysis. Administration of intravenous crystalloids, opioid, and antiemetic medications led to some clinical improvement. A low radiation protocol of noncontrast computed tomography (NCCT) of the abdomen revealed left hydronephrosis with perinephric-free fluid and accumulation of fluid in the left gutter. There were no urinary stones. To further clarify these findings, intravenous contrast was administered 3 hours later and the NCCT was completed with a computerized tomographic urography showing bilateral symmetrical renal excretion with left hydronephrosis and retroperitoneal urinary extravasation originating at the level of the left ureteropelvic junction (Fig. 1). In light of the severe extravasation and the extent of the patient's pain, we decided to drain the left kidney by means of a 7FR internal stent.

The patient denied any history of urinary calculi. She had, however, been hospitalized in our ophthalmologic department 2 weeks before the present event and was diagnosed as having acute ocular toxoplasmosis. The classical treatment with sulfadiazine, pyrimethamine, and corticosteroid was administered and she was released home with the instructions to continue on those medications for the next month.

Her clinical condition improved after intravenous fluid administration, internal ureteral stenting, and switching medications from sulfadiazine–pyrimethamine to trimethoprim–sulfamethoxazole. She was discharged symptom-free after 2 days of hospital stay with instructions to substantially increase her liquid intake as long as she continued to take the medications for the ocular toxoplasmosis. The stent was removed 4 weeks later at which time a retrograde pyelography ruled out extravasation and filling defects.

## Discussion

Precipitation and crystallization of some drugs and their metabolites in urine may cause obstructive nephropathy.^2,3^ The list of potentially crystallogenic drugs includes indinavir (a protease inhibitor), triamterene (a potassium-sparing diuretic), guaifenesin (an expectorant), ephedrine (a sympathomimetic), magnesium trisilicate (an antacid), ciprofloxacin (an antibiotic), and sulfonamides (antibiotics).^3^ Sulfadiazine is a sulfonamide used for the treatment of toxoplasmosis. Its primary metabolites include acetylated 2-sulfanilamidopyrimidine, a poorly soluble weak acid that may crystallize in urine.^4^ High-dose sulfadiazine induces crystalluria in 20%–45% of patients; however, its association with obstructive nephropathy is rare, ranging from 0.4% to 5.4% of these patients.^5,6^ The crystal deposition is enhanced by dehydration, hypoalbuminemia, chronic kidney disease, inadequate high doses, and acidic urinary pH (<5.5). Therefore, treatment involves cessation of the sulfadiazine with possible switching to a trimethoprim–sulfamethoxazole regimen, aggressive volume repletion, and urine alkalization at a pH of >7.15.^1^ Restarting sulfadiazine is not contraindicated if good hydration and urine alkalinization are maintained.

The crystals may be identified in the urinary sediment examination in shapes of needles or rosettes. Sulfa-induced calculi are pure radiolucent on plain radiographies, whereas the efficacy of their ultrasonographic identification is controversial.^3^ Several case reports have shown that NCCT may detect these stones despite their very low attenuation coefficient (<120 Hounsfield units).^4^

The ability of a low-dose NCCT protocol in identifying sulfonamide-induced stones in the urinary system has not been previously assessed. In our case, the low-dose NCCT had not revealed any urinary stones. The contrast medium was not administered until 3 hours after the NCCT, and we believe that the crystals could have already been passed or they could have already been dissolved during the time in which the patient was intravenously hydrated. The negative history of our patient for urinary calculi and the close association between the beginning of sulfadiazine therapy and the occurrence of the renal colic strongly support the diagnosis of sulfadiazine-induced obstructive nephropathy, despite the failure to detect calculi on the low-dose NCCT.

## Conclusion

This case is the first description of urinary extravasation related to obstructing sulfonamide crystals. It has been reported that sulfadiazine medication may cause a variety of nephropathies related to obstruction. Our patient presented with a severely painful acute clinical manifestation with urinary extravasation, although the symptoms can sometimes be mild and nonspecific. We believe that our case contributes to a heightened awareness of toxoplasmosis medication-induced nephropathies and alerts to its prompt recognition and appropriate treatment.

## Abbreviation Used

NCCT: noncontrast computed tomography
